# Applications of Chipless RFID Humidity Sensors to Smart Packaging Solutions

**DOI:** 10.3390/s24092879

**Published:** 2024-04-30

**Authors:** Viviana Mulloni, Giada Marchi, Andrea Gaiardo, Matteo Valt, Massimo Donelli, Leandro Lorenzelli

**Affiliations:** 1 Center for Sensors and Devices, Fondazione Bruno Kessler, 38123 Trento, Italy; gmarchi@fbk.eu (G.M.); gaiardo@fbk.eu (A.G.); mvalt@fbk.eu (M.V.); lorenzel@fbk.eu (L.L.); 2 Department of Civil Environmental and Mechanical Engineering, University of Trento, 38123 Trento, Italy; massimo.donelli@unitn.it

**Keywords:** chipless RFID, humidity sensing, smart packaging, microwave sensor, threshold sensor

## Abstract

Packaging solutions have recently evolved to become smart and intelligent thanks to technologies such as RFID tracking and communication systems, but the integration of sensing functionality in these systems is still under active development. In this paper, chipless RFID humidity sensors suitable for smart packaging are proposed together with a novel strategy to tune their performances and their operating range. The sensors are flexible, fast, low-cost and easy to fabricate and can be read wirelessly. The sensitivity and the humidity range where they can be used are adjustable by changing one of the sensor’s structural parameters. Moreover, these sensors are proposed as double parameter sensors, using both the frequency shift and the intensity variation of the resonance peak for the measure of the relative humidity. The results show that the sensitivity can vary remarkably among the sensors proposed, together with the operative range. The sensor suitability in two specific smart packaging applications is discussed. In the first case, a threshold sensor in the low-humidity range for package integrity verification is analyzed, and in the second case, a more complex measurement of humidity in non-hermetic packages is investigated. The discussion shows that the sensor configuration can easily be adapted to the different application needs.

## 1. Introduction

As an alternative to traditional packaging, smart packaging today is a smart tool capable of interacting with the environment and creating a link with manufacturers and consumers while its functions far exceed the purpose of simply containing products [1]. Its market trend is continuously growing, and it is already estimated to be worth around several billion US dollars. The uses of smart packaging are widespread, from logistics and product traceability to safety control and waste reduction [2].

In particular, the food packaging market has been deeply transformed by the introduction of smart packaging [3]. This new technology can provide in real time important information on food products, reducing the amount of waste and the health risks. To implement these functions, internal or external electronic sensors and radio frequency identification (RFID) devices are usually exploited [4]. Electronic sensors can give accurate data for different kinds of parameters observed in food products, as in the case of electronic noses and electronic tongues. These types of sensors can sense both chemical and biological parameters and are rather selective and precise. However, their size and cost make them hard to embed in packaging, especially compared with the simplicity and low cost of a barcode as in traditional packaging solutions. Moreover, the typical electronic sensor needs to be powered, and this means that it has to be either wired or provided with a battery. Both approaches are difficult and expensive to implement in an efficient and low-cost food packaging solution.

On the other hand, RFID technology has progressed significantly in the past few years, and its identification and traceability capabilities make a major contribution to food safety and quality monitoring [5]. However, to implement smart packaging functions, the outstanding tracking functions typical of RFID systems must be coupled with sensing functionalities [6] to detect environmental parameters, such as the temperature, humidity and chemical composition, to address food quality and the risk of product deterioration. RFID tags can, in principle, integrate several types of sensors, providing identification and sensing in a contactless, wireless, passive and non-visual way at the same time, but this field of research is still in the explorative stage [5]. The usual approach to coupling identification and sensing functions is to monitor the impedance of the sensor, which depends on the parameter being sensed, together with the digital data from an IC chip, which are measured with a digital RFID reader. However, this complicates the reading system, and the two functions are often measured separately [6]. Passive RFID sensors require electromagnetic power to read and write the data stored on the chip. This power comes from the RFID reader and determines the reading range in UHF tags. This range decreases with the integration of a sensing device and is one of the most important limitations of RFID sensors. Chip-based RFID sensors guarantee high accuracy in the reading process but increase the complexity and cost of the RFID tag, making it less robust and convenient for integration in the package.

As an alternative and emerging solution, chipless RFID tags [7] have been proposed in the literature by several authors. The important advantage of this new technology is the absence of integrated circuits or chips in the tag. This important feature leads to simpler, low-cost, more robust and less power-demanding devices [8] compared with more traditional RFID technologies. Moreover, chipless RFID tags have better functionality in harsh environments and can have their support, like barcode tags, printed on using high-conductivity inks [9]. The tag structure is composed of several resonating structures, and much research is available for the design and encoding [10,11] strategies of the identification (ID) function in chipless RFIDs, as well as for the detection techniques. Given the proposed application, in this study, we focus on tags called backscattered chipless RFID tags, where the resonant elements provide the information directly through their spectral signatures, as opposed to retransmission-based tags [12], which require a transmitting and a receiving antenna or a microstrip line. The backscattered tags do not need additional elements; hence, the size of the tag is strongly reduced because it contains only the resonators.

While important results have been obtained for the coding capacity of the tag and maximization of the information density in chipless RFIDs for tracking and product identification applications [13], the integration of ID [14] and sensing functionalities [15] is still a topic of active and explorative research. The two functions are usually embedded in the structure of the tag, which is composed of a series of different resonators, some of which are used for ID coding, while others are used for sensing [8]. Even though a large variety of chipless RFID sensors has already been demonstrated in the literature for physical, chemical and mechanical sensing of different parameters [8,16], they are intrinsically more challenging than the ID geometries and are still in the early explorative stage regarding their implementation in marketable applications. The main obstacles to commercialization are the reduced information density compared with chipped RFID solutions and the limited reading distance, which is common among completely passive systems. Both challenges are currently highly active topics in chipless RFID research and development.

Furthermore, when integrating chipless RFID sensors into real-world smart packaging, challenges such as a low cost, environmental robustness [17], compatibility and data security must be addressed effectively [18]. These barriers highlight the need for standardized solutions that can ensure reliable operation in different packaging environments while maintaining cost efficiency and accurate detection performance (e.g., high sensitivity), as well as addressing privacy issues to gain consumer confidence [19]. Despite their potential, overcoming these challenges will be essential for the successful implementation of chipless RFID sensors in smart packaging applications.

In this contribution, we report the development of a chipless low-cost humidity sensor for wireless monitoring of moisture levels within packaged goods, which can be easily enabled with additional identification capabilities, and which is capable of detecting an extensive range of RH% with high sensitivity. The suitability and adaptation of the sensor for different packaging requirements is discussed, and the optimization of the sensor characteristics for each application in terms of sensitivity and operational humidity range is presented. In particular, the maximization of the sensitivity for quite low-humidity environments is useful for the packaging of perishable goods, where low levels of internal humidity are essential for proper conservation. In this case, in order to assure the package integrity and consequently the stability of an inert atmosphere inside the package, a threshold sensor for signaling the minimum amounts of humidity is the most convenient choice. Other applications may require more elaborate solutions, needing more frequent and precise monitoring of humidity levels inside the package. In this case, the sensor should be able to measure a wide range of ambient humidity while a high sensitivity in a specific subrange is not required. Intermediate or mixed solutions are also possible, depending on the characteristics of the goods to be monitored. A strategy to tailor the sensor performances while taking into account the needs of the different possible packaging applications will be presented and discussed.

## 2. Materials and Methods

**Figure 1:**
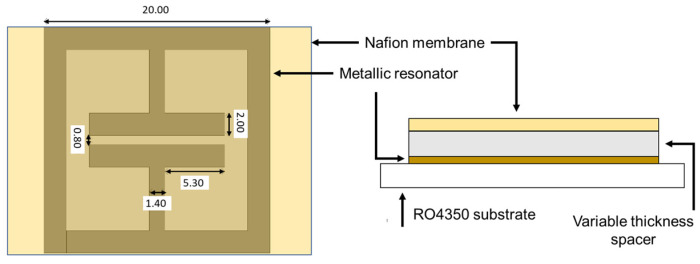
Structure and dimensions of the chipless sensors. All dimensions are in millimeters.

The sensors were composed of a metallic resonator over a flexible 168 µm-thick low-loss substrate (Rogers 4350, Rogers Corp., Chandler, AZ, USA) covered by a 50 µm-thick Nafion NRE-212 membrane. This polymer was chosen because it is extremely sensitive to environmental humidity [20]. The geometry of the metallic structure was that of a square electric field-coupled (ELC) resonator [21,22] with a lateral dimension of 20 mm and was realized by microlithography and chemical etching. The geometry of the sensing structure was simulated and described in detail in [21,23], and it is schematically reported in Figure 1.

The measurements were performed on four samples with four different distances between the metallic surface and the Nafion membrane, namely 0 (contact), 150, 400 and 800 µm. The distance was determined using plastic spacers of low-loss material (polycarbonate) of a known thickness which were transparent to the electromagnetic field. The total thickness was additionally verified with a micrometer. The RF characterization was performed by using a dedicated apparatus with a custom-made gas-flow test chamber coupled with an Agilent E5061B ENA vector network analyzer. Gas sensing measurements were performed at room temperature (21 °C). The relative humidity (%RH) was monitored by a digital humidity sensor (Honeywell HIH-4000, 1.0% accuracy) at the chamber’s exit. Here, %RH control was achieved by injecting a fixed fraction of the total dry air, namely 200 sccm (20% O_2_ and 80% N_2_), through mass flow controllers (MKSs) into a gas bubbler filled with deionized water to create the desired humidity conditions.

**Figure 2:**
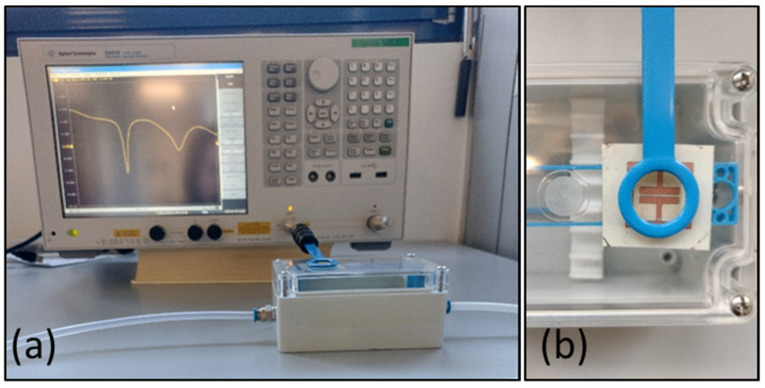
Set-up used for the measurements. (**a**) Measurement configuration showing the circular antenna probe connected to the vector network analyzer above the measurement chamber where the humidity is controlled. (**b**) Detailed image of the measurement chamber, showing the probe outside the chamber and the sensor inside the chamber.

The circular antenna probe [9], with an internal diameter of 20 mm, was directly connected to the VNA outside the chamber while the sensing tag was inside, and the detection took place wirelessly, measuring the antenna return loss S11. In particular, the antenna was a commercial magnetic field probe, namely a model H20 magnetic probe (Signal Hound, Battle Ground, WA, USA) with a frequency band from 30 KHz up to 6 GHz. The probe presented a self-resonance value of around 1.36 GHz. The distance between the tag and the probe was 0.5 cm. The measurement set-up is shown in Figure 2.

## 3. Results and Discussion

The sensors investigated in this work were dual sensors, which means that two independent sensor parameters, the peak frequency and peak intensity, varied with the analyte concentration. A third parameter, the peak width, varied as well, but it could not be considered independent from the peak intensity, being almost inversely proportional to it. This behavior is physically related to the independent variation of both the real and imaginary part of the dielectric constant of the sensing material upon exposure to humidity, where the real part variation is principally related to the frequency shift, and the imaginary part is related to the change in the peak intensity [22]. For sensing purposes, this is an advantage because the sensor can be adapted to the application-specific needs in a more versatile way, and the simultaneous determination of two parameters increases the reliability and accuracy of the sensing process. This advantage has, however, an intrinsic drawback: the peak broadening and the decreased intensity set a limit to the humidity range that can be sensed, because the more sensitive the material is, the faster the weakening of the peak signal with increasing humidity is. This behavior is material-dependent, but the trend is typically the same for all humidity-sensitive materials because they incorporate water, which has a rather high real and imaginary part of the dielectric constant as the humidity increases. At a higher humidity, the resonant peak is therefore expected to shift to lower frequencies and decrease its intensity up to a limit that makes it no longer detectable. In this work, Nafion NRE-212 was used as the sensitive material. This material is a commercial polymeric membrane that has shown a great affinity for water. Moreover, it is mechanically and chemically very resistant and stable, and its absorption and desorption of water are quite fast [21,24], making it a rather convenient sensitive material for humidity detection.

In the case of the sensors evaluated in this work, the distance between the resonator and the sensitive material was varied to explore the effect of this parameter on the detectable humidity range and on the sensitivity. This was accomplished using a spacer of a variable thickness between the metallic resonator and the Nafion membrane to adapt the sensor to different humidity ranges and detection strategies. Four spacer thicknesses were selected, as reported in the previous section.

**Figure 3:**
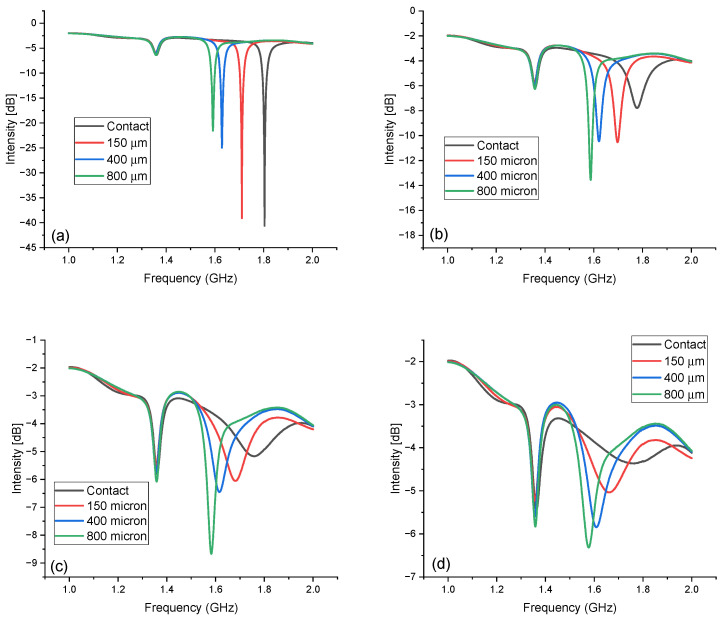
(**a**) Return loss intensity as a function of the frequency in the range of 1–2 GHz for the four resonators at (**a**) 0% RH. (**b**) 25% RH, (**c**) 50% RH and (**d**) 70% RH.

The four sensors were measured in the range of 0–70% RH between 1 and 2 GHz. The measured return loss intensities of the circular antenna probe are reported in Figure 3 at 0%, 25%, 50% and 70% relative humidity. The data are reported for the four spacer thicknesses, as indicated in the legend.

The peak intensity, bandwidth and frequency all varied markedly, going from 0 to 70% RH for all spacer values. The intensity variation is especially evident when considering the different vertical scales in the four plots of Figure 3. While in Figure 3a, the baseline signal seems almost negligible, in Figure 3d, the peak intensity is so low that the baseline signal is comparable with the peak intensities. In addition, the peak at 1.36 GHz, which was the internal resonance of the probe, did not vary appreciably with the humidity and spacer thickness and could be used to visualize the intensity variations and as a reference for sensor calibration. The intensity trend with the spacer thickness was reversed when going from 0% RH to 70% RH. The resonator with no spacer had the highest intensity at 0% RH but the lowest one at 70% RH, where it was quite broad and barely detectable. On the other hand, the 800 µm sensor had the lowest intensity at 0% RH but the highest one at 25, 50 and 70% RH. The 150 and 400 µm sensors had an intermediate behavior.

**Figure 4:**
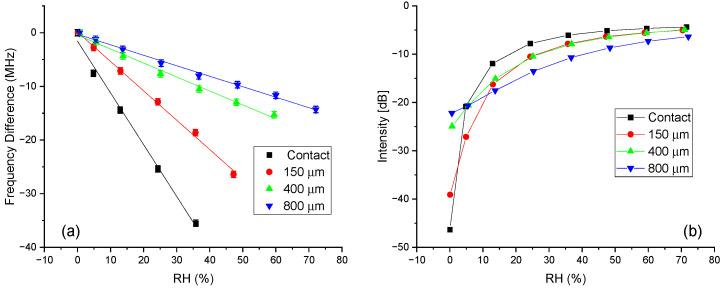
(**a**) Peak frequency variation as a function of the %RH in the range of 0–70% for the four resonators. The value at 0% RH was taken as reference. (**b**) Peak intensity as a function of the %RH in the range of 0–70% for the four resonators.

A more systematic variation of the peak intensity and frequency as a function of the %RH is reported in Figure 4. In order to better compare the different sensors, Figure 4a reports the frequency variations and not the frequency values, because the reference value of the sensors (0% RH) depended on the spacer thicknesses (see Figure 3a). Moreover, the frequency differences are shown only in the range where they had a linear trend with the %RH. It is worth noticing that the effects of the frequency shift were only on the second resonance peak, since the first peak was only due to the antenna characteristics (internal probe peak frequency). The data were plotted together with a linear fit of the experimental data, where the slope of the line represents the sensitivity of the sensor. The numerical values for the sensitivity are reported in Table 1 together with the standard error. The Pearson coefficients (R^2^) for linearity are also reported. More detailed statistics are given in Supplementary Material S1. The range of linearity of the sensors was estimated by the last measured %RH value that gave an R^2^ value larger than 0.99. Even though this is somehow an arbitrary and restrictive criterion, it should be noted that the peak broadening with increasing humidity also increased the error in the determination of the peak position, and this in turn increased the error in the sensitivity determination. The sensitivities reported in Table 1 show good values for all the spacer thicknesses, but they decreased when increasing the distance between the sensing material and the metallic resonator. The best values were given for the sensor with no spacer, where sensitivities of around 1 MHz/%RH were reached, while the sensor with a spacer of 800 µm showed a sensitivity which was about one fifth of this value. The highest values were better than those of other chipless RFID humidity sensors measured with a near-field probe [25,26]. The most sensitive microwave sensors exploit higher frequency ranges [27,28,29,30] and show higher shifts, but they require more sophisticated, large and expensive antenna systems, which are hardly suitable for applications like smart packaging. A more comprehensive list reporting the comparable existing literature is given in Table 2. For comparison, two examples of microstripped tags are also included [22,31]. They showed good performances compared with the ones measured with a near-field probe, but the detection was not wireless and required SMA connectors.

The peak intensity as a function of the %RH, reported in Figure 4b, was largest in the low humidity range, especially below 10% RH, for all the sensors investigated and did not have a linear behavior range. Below 10% RH, the peaks were quite sharp and well-defined, allowing for a very precise determination of the peak frequency. In the case of the sensor with no spacer and with the Nafion membrane in direct contact with the metallic resonator, the peak intensity and its variation were by far the largest for extremely low %RH values. In this case, when going from 0% to 5% RH, the intensity reduced to less than half, and the frequency shift was around 5 MHz. However, at higher %RH values, the intensity drop was less evident, and at approximately 20–30% RH, the signal approached a plateau, and the additional variation became difficult to detect.

It should be noted that the values reported in Figure 4b include the baseline signal that can be estimated at approximately 3–4 dB, which means that at %RH values greater than 20%, the peak intensity signal was comparable with the baseline or less. The other three sensors had a similar behavior, but their detectable %RH range was larger and increased when increasing the spacer thickness. However, the corresponding drop at low %RH values also decreased. Furthermore, when comparing the intensity variation of the sensors reported in this work with those in Table 2, it is clear that the contact sensor reported in this study showed an intensity variation that exceeded all the other chipless sensors listed. This specificity enables the use of the intensity variation as the primary detection parameter or, alternatively, the use of both the frequency shift and intensity variation as sensing parameters to improve the reliability and sensitivity of the detection. Both of these approaches will be discussed in detail in Section 3.1 and Section 3.2.

### 3.1. Threshold Humidity Sensor

The high sensitivity of the chipless RFID sensors in the low humidity range, coupled with remote signal detection, makes it particularly suitable for smart packaging integrity control in the case of a dry or inert atmosphere inside the package, which is common in the case of perishable goods. The remote monitoring proposed allows for verification of the integrity status of the package’s internal atmosphere in a totally noninvasive manner even with non-transparent packages. Moreover, the simple integrity check does not require a precise determination of the humidity but only the detection of a value above a threshold. This can easily be determined by comparing the intensity at the reference frequency of the internal probe with the reference peak frequency (0% RH) of the sensor, requiring only a comparison of the signal at two discrete frequencies, greatly simplifying the detection mechanism and consequently the cost and complexity of the reader.

**Figure 5:**
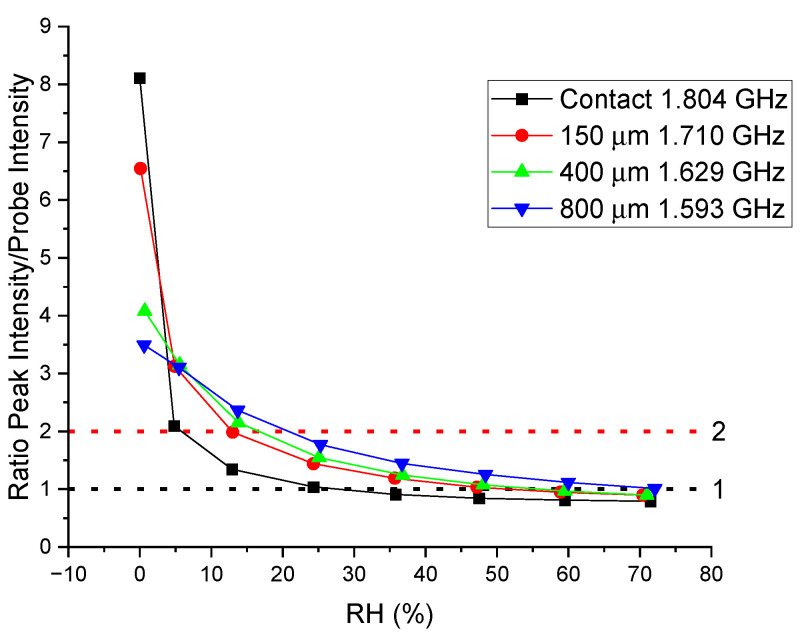
Intensity ratios of the reference peak frequency (0% RH) and the internal probe peak frequency for the four resonators investigated. The probe frequency is 1.360 GHz for all and does not vary with the humidity or spacer thickness of the sensor.

In Figure 5, the ratio between the intensity of the peak frequency at 0% RH and the probe peak intensity at 1.360 GHz is reported for all four resonators. The dashed horizontal lines are a guide to detect the %RH value, where this ratio is equal to one (black line) or two (red line). The trend is similar to what is reported in Figure 4b, but the ratio at a fixed frequency decayed faster with increasing humidity compared with the peak intensity because the effect of the peak shift added to the resonance broadening. The frequency and intensity of the probe peak remained remarkably constant within the experimental error of the measurement.

As can be seen from Figure 5, the ratio equal to one was reached only at a high humidity for all the sensors, with the sensor without a spacer crossing the black line at approximately 30%. The ratio equal to two is preferable because it gave a better differentiation among the sensors and a sharper drop near the threshold. The sensor with no spacer crosses the red line at 5%, the 150 µm sensor at 12%, the 400 µm sensor at 15% and the 800 µm sensor near 20% RH.

In order to have a threshold sensor capable of verifying the integrity of a package with an internal dry atmosphere, the sensor without a spacer can be the best choice because of its extremely high intensity drop and its low %RH threshold value. However, plastic packages are never totally hermetic, and a 5% humidity threshold could be too low because the sensor can detect a package integrity break when this is not real. Considering these tolerances, which depend on the specific package material and requirements, a sensor with a thin spacer can be, in this case, a more reasonable choice because the intensity drop is still high, but the threshold humidity can be tuned to higher %RH values.

### 3.2. Measurement of %RH inside the Package

When the purpose of smart packaging is not only to verify the package integrity but also to give precise information about the level of humidity inside the package, a threshold sensor is not enough, and a more accurate reading of the sensor signal is required. This may be the case for a non-hermetic package or packaged food that produces humidity during the degradation process. In this last case, a high humidity level may be an important indication of the food preservation status. In these conditions, the reading can still be made only at two discrete frequencies to reduce the detection complexity, but it must be much more precise, and the strong nonlinearity of the sensor response is an important problem because the sensitivity varies considerably with the %RH. This variation was extremely high for the contact sensor (%RH range: 0–36%) and the 150 µm sensor (%RH range: 0–47%). Therefore, the thicker spacer sensors are preferable for the usual ambient humidity values, which are seldom below 20%.

**Figure 6:**
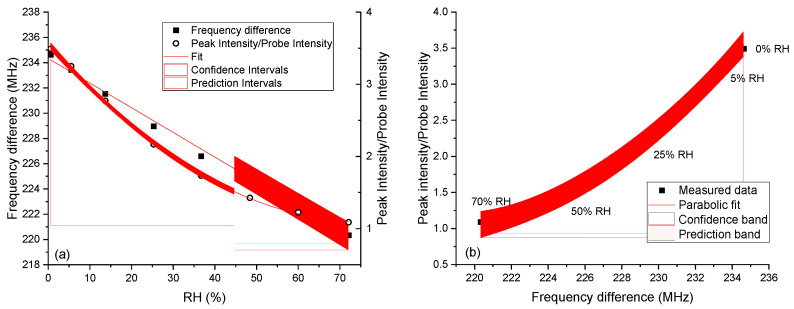
(**a**) Frequency difference (linear fit) and intensity ratio (parabolic fit) for the resonance peak of the 800 µm sensor relative to the probe peak at 1.36 MHz as a function of the %RH. (**b**) Correlation between frequency difference and intensity ratio (parabolic fit).

For improving the accuracy, the measurement of the frequency shift, which varies linearly, may provide a more reliable determination of the %RH levels compared with the intensity change, even if it needs a more complex reading system. The sensor without a spacer and the 150 µm sensor had the best sensitivities, but they were linear only up to 36 or 47 % RH. They are therefore the best choice only up to these humidity values. If a wide humidity range is required, then sensors with thick spacers must be used. In particular, the 800 µm sensor offered the widest range of linearity (%RH range: 0–70%) for the frequency variation, and its intensity trend approached linearity in the same range as well (see Figure 4b), indicating that the sensitivity did not vary much with the %RH for this parameter. This gives the opportunity to fully exploit the dual parameter detection, increasing the reliability and accuracy of the %RH determination. This not only improves the overall measurement error but can also prevent large errors due to incorrect calibration or sensor malfunctioning because it requires consistency between the peak frequency and intensity. In this case, the probe’s internal resonance can be used as a reference for both the frequency and the intensity. The frequency difference between the resonator peak and the probe peak and their intensity ratio are reported in Figure 6, together with the correlation between the two parameters.

Together with the data fit, the intervals where the mean %RH value is located (confidence) or the future measurement value is located (prediction) with 95% probability are also reported. As expected, the frequency difference can be conveniently fitted with a linear trend, while the intensity ratio requires a parabolic fit. However, as can be seen from the confidence intervals in Figure 6a, the measurement of the intensity was more accurate than the frequency in the whole humidity range investigated. The fit parameters and the relevant statistics for the fitting functions are reported in Supplementary Material S1.

## 4. Conclusions

In this paper, we proposed the use of chipless RFID humidity sensors using Nafion 212 as the sensitive material for smart packaging of food items. A new methodology for tailoring the operating range of the sensor was presented based on the variation of the distance between the metallic resonator and the sensing material. Four sensors were proposed with different distances between the sensing material and the metallic resonator. Moreover, given the specificity of the detection mechanism, the proposed detection methodology can make use of two sensing parameters: the peak frequency shift and peak intensity variation. It was then shown how the combined use of the two parameters made the detection more precise and reliable.

Two different sensor applications were examined. In the first case, a threshold sensor for a package with a dry or controlled internal atmosphere was considered. In this case, a sensor able to detect extremely low humidity levels was the best choice to verify the package integrity, but an accurate determination of the %RH was not necessary. We found that the sensor with the sensing material in direct contact with the resonator showed an extremely high intensity variation at low humidity values and was therefore especially suited for this application. In this specific case, the detection at one or two frequencies was enough for integrity verification, and consequently, the reading system could be simplified.

In the second case, a non-hermetic package was examined. For this application, a more exact determination of the humidity is usually required over a wider span of %RH values. The best solution in this case was the sensor with a spacer of 800 µm. This sensor was worse in terms of frequency variation but covered a wider range of humidity. Moreover, it allowed the exploitation of two sensing parameters, the peak frequency and intensity, with the same measurement, increasing the reliability and accuracy of the %RH determination. Although the capabilities of the chipless RFID sensor developed in this work for the detection of different RH% values were successfully demonstrated, it is important to highlight that a laboratory-assembled configuration was required to perform the detection tests, which cannot be used as is for real applications. For the effective implementation of these sensors in smart packages, further research is needed, especially for chipless sensor signal reading technology, and this will be one of the directions for future work.

## Figures and Tables

**Table 1:** Sensitivity (MHz/%RH) of the investigated sensors.

Spacer Thickness (µm)	Sensitivity (MHz/%RH)	Error (Standard Deviation)	Relative Error (%) ^1^	Pearson Square (R^2^)	Humidity Range (%)
0	−0.965	0.044	4.6%	0.99375	0–36
150	−0.547	0.014	2.5%	0.99756	0–47
400	−0.260	0.011	4.1%	0.9917	0–60
800	−0.195	0.006	2.8%	0.99529	0–72

^1^ Calculated as the standard deviation/sensitivity ratio (%).

**Table 2:** Performance comparison of chipless sensors for humidity detection from the literature.

Ref.	Substrate	Sensitive Material	Tag Size (cm^2^)	Resonator	Reading Range	%RH Range	Frequency Range (GHz)	Frequency Shift	Intensity ^1^ Variation
[25]	Photopaper	Poly-vinyl alcohol (PVA)	-	Interdigitated LC	2 cm: Near-field probe (NFP)	86–91	0.128–0.132	1.2 KHz/%RH	0.014 dB/%
[32]	Taconic TLX 0	PVA	1.5 × 0.68	ELC	30 cm: horn antennas	35–85	6–7	12 MHz/%RH	0.02 dB/%
[27]	Kapton HN	Substrate	5.4 × 5.4	Van-Atta reflectarray	5.5 m: horn antennas	10–75	25–35	40 MHz/%RH	Not reported
[29]	Kapton HN	Substrate	7.4 × 7.4	Van-Atta reflectarray	58 m: radar	30–75	24	-	0.2 dB/%
[26]	PET, paper	Substrate	4 × 4	Interdigitated LC	0.5 cm: NFP	20–90	0.12–0.3	0.37 MHz/%RH	Not reported
[31]	FR-4	SnO_2_/graphene	5 × 2	4 C-shaped resonators	Microstrip (wired)	11–98	2.85–3.05	2.95 MHz/%RH	Negligible
[30]	Polycarbonate	Silicon nanowires	4 × 2	Rectangular loops	Not reported: horn antennas	74–98	3.295–3.33	1.5 MHz/%RH	-
[24]	DiClad	Nafion117	4 × 2	ELC	Microstrip	5–40	2.49–2.60	2.71 MHz/%RH	0.04 dB/%
This ^2^ work	Rogers 4350	Nafion 212	2 × 2	ELC	0.5 cm: NFP	0–36		0.965 MHz/%RH	1.12 dB%

^1^ Average value over the %RH range reported. ^2^ Values for the contact sensor.

## Data Availability

The data are available upon request from the corresponding author.

## Supplementary Materials

The following supporting information can be downloaded at https://www.mdpi.com/article/10.3390/s24092879/s1. Contact Sensor: linear fit in Figure 2a, 150 µm Sensor: linear fit in Figure 2a, 400 µm Sensor: linear fit in Figure 2a, 800 µm Sensor: linear fit in Figure 2a, 800 µm Sensor: linear fit in Figure 6a, 800 µm Sensor: parabolic fit in Figure 6a, 800 µm Sensor: parabolic fit in Figure 6b.
