# Supplementary material for: Applications of Chipless RFID Humidity Sensors to Smart Packaging Solutions
Source: Sensors (Basel). 2024 Apr 30;24(9):2879. doi: 10.3390/s24092879 (PMC11086060; doi:10.3390/s24092879)
Supplement: Supplementary file 1 [file sensors-24-02879-s001.zip › sensors-2903036-supplementary.pdf]

Supplementary Information

Contact Sensor: linear fit in Figure 2(a)

Equation	y = a + b*x
Plot	Freq. Difference
Weight	No Weighting
Intercept	-0.00156 ± 8.96617E-1
Slope	-9.64606E-1 ± 4.41562E-2
Residual Sum of Squares	4.97783E
Pearson's r	-0.99687
R-Square (COD)	0.99375
Adj. R-Square	0.99167

150 μm Sensor: linear fit in Figure 2(a)

Equation	y = a + b*x
Plot	Freq. Difference
Weight	No Weighting
Intercept	1.14508E-1 ± 3.61396E-1
Slope	-5.47126E-1 ± 1.3522E-2
Residual Sum of Squares	1.22384
Pearson's r	-0.99878
R-Square (COD)	0.99756
Adj. R-Square	0.99695

400 μm Sensor: linear fit in Figure 2(a)

Equation	y = a + b*x
Plot	Freq. Difference
Weight	No Weighting
Intercept	-4.46794E-1 ± 3.61397E-1
Slope	-2.60231E-1 ± 1.06501E-2
Residual Sum of Squares	1.65882
Pearson's r	-0.99584
R-Square (COD)	0.9917
Adj. R-Square	0.99003

800 μm Sensor: linear fit in Figure 2(a)

Equation	y = a + b*x
Plot	Freq. Difference
Weight	No Weighting
Intercept	-3.00176E-1 ± 2.24114E-1
Slope	-1.95428E-1 ± 5.487E-3
Residual Sum of Squares	0.857346
Pearson's r	-0.99764
R-Square (COD)	0.99529
Adj. R-Square	0.99451

800 µm Sensor: linear fit in Figure 6(a)

Equation	y = a + b*x
Plot	Frequency difference
Weight	No Weighting
Intercept	234.33547 ± 0.22411
Slope	-0.19543 ± 0.00549
Residual Sum of Squares	0.85735
Pearson's r	-0.99764
R-Square (COD)	0.99529
Adj. R-Square	0.99451

800 µm Sensor: parabolic fit in Figure 6(a)

Equation	y = Intercept + B1*x^1 + B2*x^2
Plot	Intpeak/Intprobe
Weight	No Weighting
Intercept	3.56044 ± 0.02178
B1	-0.06493 ± 0.00155
B2	4.27552E-4 ± 2.11675E-5
Residual Sum of Squares	0.00409
R-Square (COD)	0.99933
Adj. R-Square	0.99906

800 µm Sensor: parabolic fit in Figure 6(b)

Equation	y = Intercept + B1*x^1 + B2*x^2
Plot	Intpeak/Intprobe
Weight	No Weighting
Intercept	395.61488 ± 51.24065
B1	-3.63683 ± 0.45016
B2	0.008389 ± 88329E-4
Residual Sum of Squares	0.01438
R-Square (COD)	0.99763
Adj. R-Square	0.99668
